# The Effects of Metformin on the Gut Microbiota of Patients with Type 2 Diabetes: A Two-Center, Quasi-Experimental Study

**DOI:** 10.3390/life10090195

**Published:** 2020-09-11

**Authors:** Hanako Nakajima, Fumie Takewaki, Yoshitaka Hashimoto, Shizuo Kajiyama, Saori Majima, Hiroshi Okada, Takafumi Senmaru, Emi Ushigome, Naoko Nakanishi, Masahide Hamaguchi, Masahiro Yamazaki, Yoshiki Tanaka, Yousuke Oikawa, Shunji Nakajima, Hiroshi Ohno, Michiaki Fukui

**Affiliations:** 1 Department of Endocrinology and Metabolism, Graduate School of Medical Science, Kyoto Prefectural University of Medicine, Kyoto 602-8566, Japan; tabahana@koto.kpu-m.ac.jp (H.N.); fumi12112000@yahoo.co.jp (F.T.); kajiyama-clinic@dream.ocn.ne.jp (S.K.); saori-m@koto.kpu-m.ac.jp (S.M.); conti@koto.kpu-m.ac.jp (H.O.); semmarut@koto.kpu-m.ac.jp (T.S.); emis@koto.kpu-m.ac.jp (E.U.); naoko-n@koto.kpu-m.ac.jp (N.N.); mhama@koto.kpu-m.ac.jp (M.H.); masahiro@koto.kpu-m.ac.jp (M.Y.); michiaki@koto.kpu-m.ac.jp (M.F.); 2 Kajiyama Clinic, Kyoto 600-8898, Japan; 3 Department of Internal Medicine, Matsushita Memorial Hospital, Moriguchi 570-8540, Japan; 4 R&D Center, Biofermin Pharmaceutical Co., Ltd., Kobe 650-0021, Japan; tanaka_yoshiki@biofermin.co.jp (Y.T.); oikawa_yousuke@biofermin.co.jp (Y.O.); nakajima_shunji@biofermin.co.jp (S.N.); ohno_hiroshi@biofermin.co.jp (H.O.)

**Keywords:** metformin, diabetes mellites, dysbiosis, gastrointestinal symptoms

## Abstract

Metformin is reported to affect human gut microbiota; however, the nature of this association in Japanese patients with type 2 diabetes mellitus (T2DM) is unknown. We enrolled 31 patients with T2DM who took metformin for the first time in this study. We compared them before and after four weeks of taking metformin. Fecal samples were collected and 16S rDNA sequences were performed to identify the gut microbiota. Blood samples and Gastrointestinal Symptom Rating Scale (GSRS) questionnaire results, denoting gastro-intestinal symptoms, were also collected. In the whole-group analysis, no significant differences were found at the phylum level. In a subgroup of 21 patients that excluding those using medications affecting gut microbiota, there was a significant decrease of the phylum Firmicutes (*p* = 0.042) and of the ratio of the Firmicutes and Bacteroidetes abundances (*p* = 0.04) after taking metformin. Changes in abdominal pain (*r* = −0.56, *p* = 0.008) and regurgitation (*r* = −0.53, *p* = 0.01) were associated with *Parabacteroides*. Despite there being no direct association with abdominal symptoms, our study revealed that the composition of gut microbiota in Japanese individuals with T2DM partially changed after starting metformin.

## 1. Introduction

Type 2 diabetes mellitus (T2DM) has become a major public health concern worldwide [1]. The study of gut microbiota is rapidly progressing and it is known that the balance of the gut microbiota is disordered in patients with T2DM, which is a condition known as dysbiosis [2]. Recent studies have reported that the various medications used for T2DM affect gut microbiota [3,4,5,6]. 

Metformin has been used as the first-line drug for the medication of T2DM, especially in Western countries, because of its price, safety, and protective effects on cardiovascular disease (CVD) mortality [7,8]. Metformin performs several actions within the gut. It increases intestinal glucose uptake and lactate production, increases glucagon-like peptide 1 (GLP-1) concentrations and the bile acid pool within the intestine, and alters the microbiota [9,10]. This evidence suggests that metformin may also have a synergistic effect with the gut microbiota. Thus, the effect of metformin on gut microbiota has been a primary focus [11,12,13]. However, existing studies on the effects of metformin on gut microbiota have been carried out in Western countries and China [11,13], and as such, we know that the gut microbiota’s characteristics vary between countries, including in patients with T2DM [14,15]. Thus, the effect of metformin on the gut microbiota of Japanese people with T2DM is still unclear, and this fact has motivated the present research. Therefore, the main purpose of this study was to clarify the change of gut microbiota after taking metformin in Japanese patients with T2DM. Moreover, regardless of the illness duration, patients with T2DM are known to suffer from a high prevalence of abdominal symptoms due to dysbiosis [16]. It has been reported that metformin increases abdominal symptoms, such as diarrhea, constipation, and gastrointestinal (GI) discomfort [17,18]. The Gastrointestinal Symptom Rating Scale (GSRS) is a useful and widely used questionnaire [19,20,21] and can quantitatively evaluate a patient’s quality of life (QOL) based on the GI symptoms experienced by the patient [22,23]. Using this scale, we also researched changes in GI symptoms before and after taking metformin. 

## 2. Results

### 2.1. Differences between before and Four Weeks after Medication

In this study, 20 men and 11 women were enrolled. None of the subjects withdrew from the study and no missing data for each variable were observed. The baseline characteristics of the study individuals are shown in Table 1. The mean age, body mass index (BMI), and hemoglobin A_1c_ (HbA_1c_) were 63.3 ± 9.5 years, 23.3 ± 3.2 kg/m^2^, and 55.4 ± 7.9 mmol/mol, respectively. 

**Figure 1:**
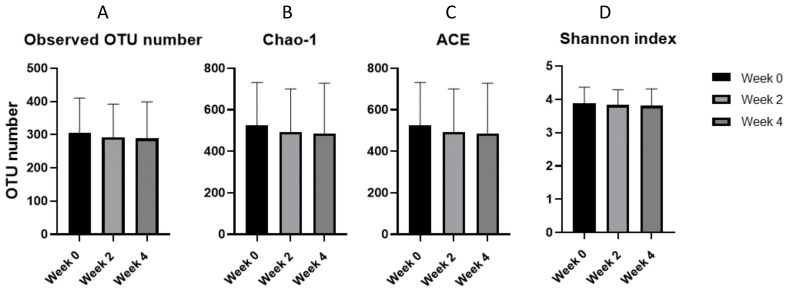
The α-diversity comparisons for each period: (**A**) observed operational taxonomic units (OTUs) number, (**B**) Chao1 index, (**C**) abundance-based coverage estimator (ACE), and (**D**) Shannon index. The differences between the groups were evaluated using paired *t*-tests, with no significant differences being found.

The α-diversity did not show a significant difference between before and after four weeks of medication (Figure 1). 

**Figure 2:**
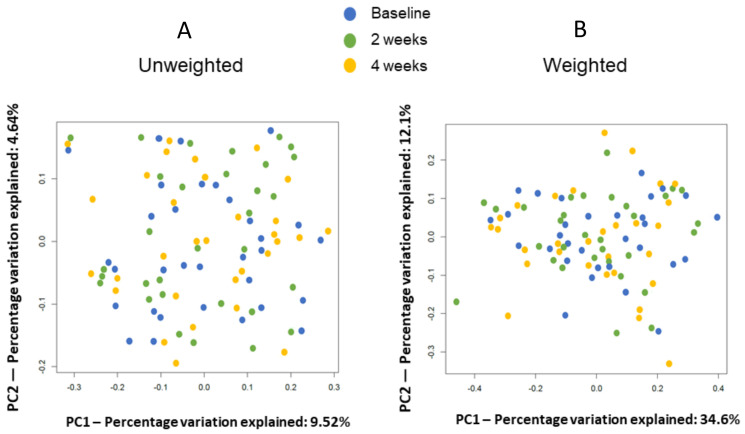
Principal coordinates analysis (PCoA) of the microbiome fecal diversity among all samples based on the duration of metformin usage: (**A**) unweighted UniFrac metrics and (**B**) weighted Unifrac metrics. There was no significant difference between the groups (evaluated using permutational multivariate analysis of variance (PERMANOVA)).

We also observed no significant differences in the β-diversity estimates (Figure 2). 

**Figure 3:**
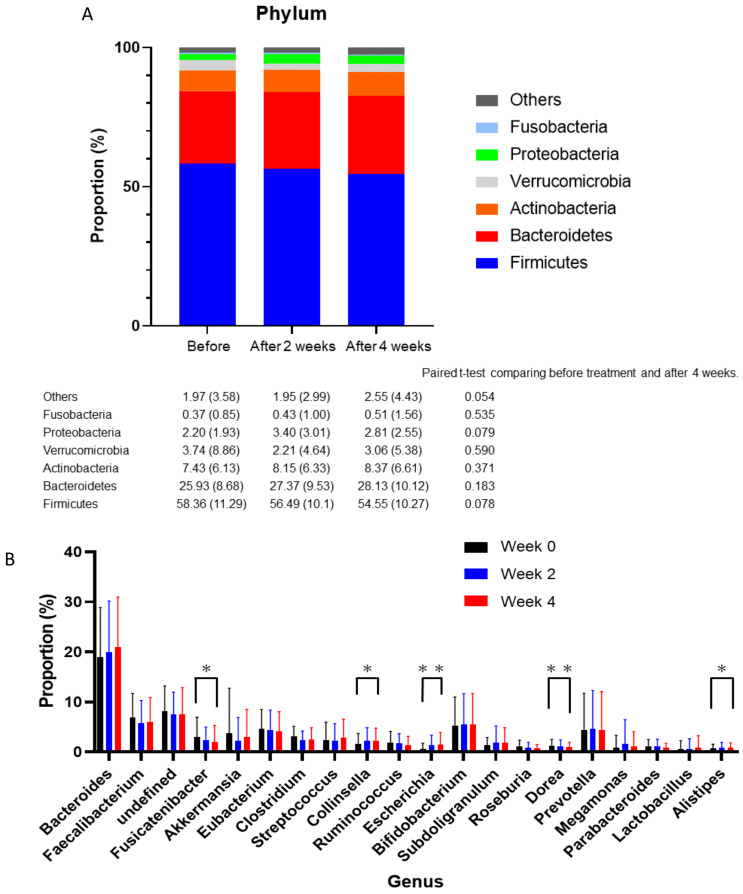
(**A**) Alternation of phyla over four weeks of taking metformin and the relative abundances of phyla. Paired *t*-tests were performed to evaluate the phyla before and after four weeks of medication. (**B**) The weighted average difference (WAD) method was used for detecting differentially expressed genes in all participants. The top 20 most abundant gut microbial genera are shown and the differences between these genera were evaluated using paired *t*-tests. * *p* ≤ 0.05, ** *p* ≤ 0.01.

At the phylum level, subtle increases of Bacteroidetes and Actinobacteria, and a decrease of Firmicutes, were observed, though these were not statistically significant (Figure 3A). In addition, the ratio of Firmicutes to Bacteroidetes decreased after four weeks of taking metformin, although this was not statistically significant (before: 2.82 ± 1.99; after two weeks: 2.50 ± 1.44; after four weeks: 2.39 ± 1.47; *p* = 0.22).

The top 20 most abundant genera, as determined using the weighted average distance (WAD) method, are shown in Figure 3B. According to the linear discriminant effect size (LEfSe) analysis, no genera change was observed.

### 2.2. Subanalysis Excluding Medications that Affect Gut Microbiota

**Figure 4:**
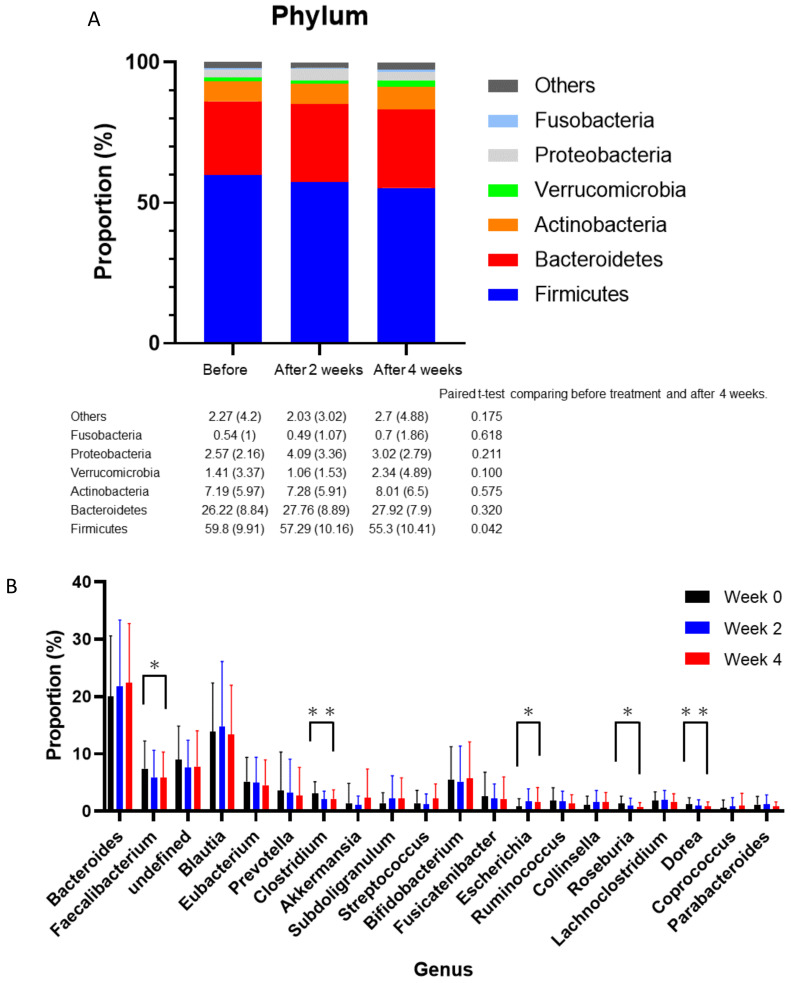
(**A**) Alternation of gut microbiota over four weeks of taking metformin in the subgroup and relative abundances of the phyla. Paired *t*-tests were performed to evaluate the gut microbiota before and four weeks after medication. (**B**) The WAD method was used for detecting differentially expressed genera in the subgroup. The top 20 gut microbial genera are shown and differences among these genera were evaluated using paired *t*-tests. For the subgroup analyses, the participants who used an α-glucosidase inhibitor, a dipeptidyl peptidase IV inhibitor, a proton pump inhibitor, or an H2 blocker were excluded.

**Figure 5:**
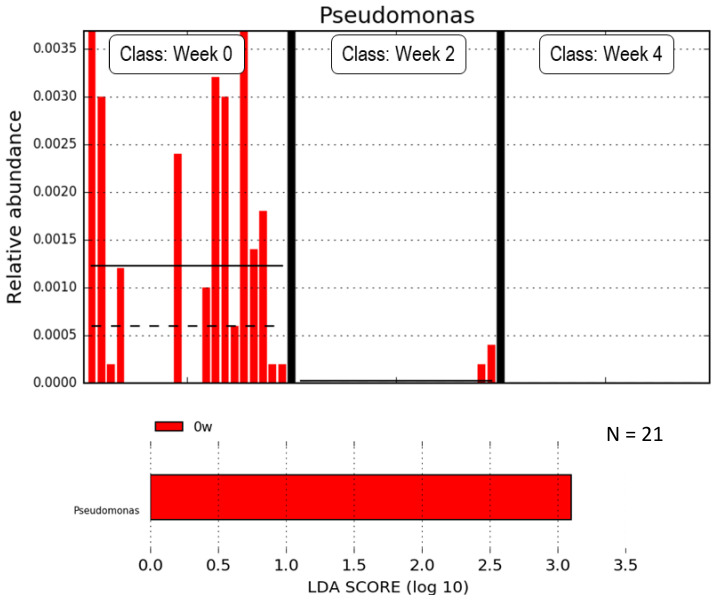
Linear discriminant analysis (LDA) in the subgroup showing the histogram of the Pseudomonas relative abundances for each period. The amount of *Pseudomonas* decreased after taking metformin.

Next, we excluded participants taking medications that may affect their gut microbiota. The clinical characteristics of this group are presented in Table 1. Metformin altered the gut microbiota composition during the study period in this subgroup. At the phylum level, the abundance of Firmicutes decreased when metformin was taken (*p* = 0.042) (Figure 4A). In addition, the ratio of the Firmicutes and Bacteroidetes abundances decreased four weeks after taking metformin (before: 2.72 ± 1.45; after two weeks: 2.42 ± 1.28, after four weeks: 2.26 ± 1.09; *p* = 0.04). The top 20 most abundant genera in the subgroup according to the WAD method are shown in Figure 4B. According to the LEfSe analysis, the genus *Pseudomonas* strongly decreased when subjects took metformin (Figure 5). 

### 2.3. Relationships between the GSRS and Gut Microbiota in the Subanalysis

We examined the differences in blood composition, GSRS, and Bristol Stool Form Scale before and after participants received the metformin (Table 2). In both groups, glycemic control was clearly improved by the metformin (*p* = 0.0003 in the whole group; *p* = 0.0005 in the subgroup). The severity of dyspepsia and constipation was found to be high at the baseline of this study. After four weeks of receiving the medication, constipation was more severe (baseline: 4.7 ± 1.9; after four weeks: 5.9 ± 2.3; *p* = 0.03) in participants than diarrhea (baseline: 3.1 ± 1.4; after four weeks: 3.3 ± 1.7; *p* = 0.83).

**Figure 6:**
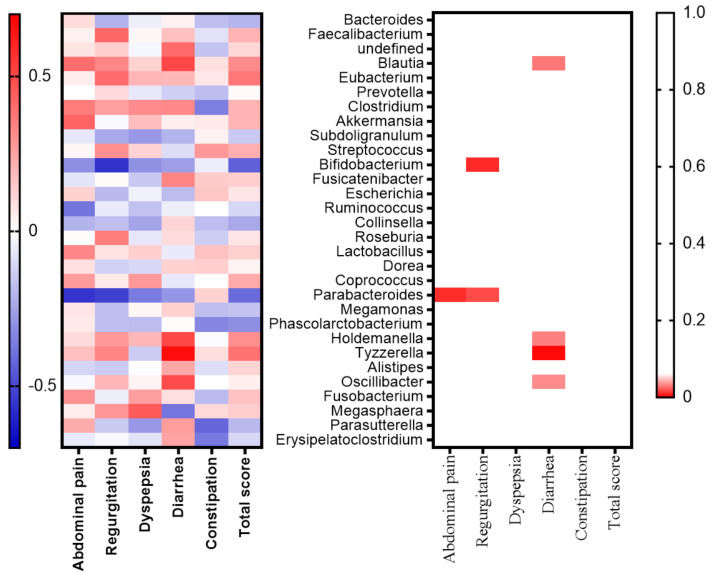
Heat map showing changes in the relative abundance of genera after the metformin treatment in the subgroup. The association between the changes in gastrointestinal symptoms and changes in the relative abundance of genera before and after four weeks of medication were compared. The top 20 genera are shown along the y-axis and the gastrointestinal symptoms are shown along the x-axis. The left panel shows the correlation coefficients. Red denotes a positive association and blue denotes a negative association (Spearman’s correlation coefficient). The right panel shows the *p*-values (Spearman’s correlation coefficient). Red indicates a *p*-value < 0.05.

Lastly, we investigated which genera contributed to the deterioration or improvement of each abdominal symptom in the subgroup. As shown in Figure 6, there was a significant relationship between abdominal pain and *Parabacteroides* (r = −0.56, *p* = 0.008). Regurgitation was found to be associated with a decrease in *Parabacteroides* (r = −0.53, *p* = 0.01) and *Bifidobacterium* (r = −0.56, *p* = 0.008). Diarrhea was associated with an increase in *Tyzzerella* (r = 0.66, *p* = 0.001), *Blautia* (r = 0.50, *p* = 0.02), *Holdemanella* (r = 0.5, *p* = 0.03), and *Oscillibacter* (r = 0.49, *p* = 0.03). Symptoms of constipation had no association with specific genera.

## 3. Discussion

This study investigated the changes in gut microbiota in Japanese patients with T2DM after taking metformin and the changes in GI symptoms before and after taking metformin and its association with gut microbiota. This study showed that there were no significant changes in phyla or genera due to the metformin usage for all participants, as shown in the PCoA plots, relative abundance plots, and LEfSe analysis results. One of the reasons for this was that the duration of metformin usage was short and the changes in the gut microbiota may not have been adequately observed. Another reason may have been that the other medications had a strong impact on the gut microbiota. In fact, in a subanalysis of patients who were not taking medications that might affect their gut microbiota, there was a significant difference in the relative abundance plots, WAD, and LEfSe before and after the metformin usage.

In this study, the baseline gut microbiota in T2DM Japanese patients before medication did not differ from the previous reports. In fact, the proportion of phylum Bacteroidetes abundance was low and that of phylum Firmicutes was high, which are characteristic of patients with type 2 diabetes [5,24,25,26]. However, in this study, the genera *Bacteroides* and *Escherichia* had positive correlations with metformin usage and the genera *Faecalibacterium* and *Ruminocococus* had negative correlations with metformin usage. Thus, it can be said that metformin had a partial effect on the gut microbiota in Japanese patients with T2DM.

On the other hand, we revealed that the ratio of the Firmicutes and Bacteroidetes abundances significantly decreased in the subgroup after four weeks of taking metformin. The genus *Bacteroides* (phylum Bacteroidetes) increased and the genus *Faecalibacterium* (phylum Firmicutes) decreased after medication. Regarding the changes in other genera, in the phylum Firmicutes, we observed a reduced abundance of *Clostridium*, as has been previously reported [11]; *Roseburia* and *Dorea* (phylum Firmicutes) also decreased after medication. Although no significant change in body weight was observed in this study, it has previously been reported that the ratio of the Firmicutes and Bacteroidetes abundances is high in obese people [27].

The genus *Pseudomonas*, which is typified by its high biofilm-forming capacity, decreased in abundance after taking metformin, as shown in the subgroup analysis. We considered that metformin could cause a significant decrease in biofilm formation due to its anti-bacterial activity, which might result in a significant decrease in *Pseudomonas aeruginosa* after medication. Recent studies similarly revealed that metformin prevents and regulates the effects of *P. aeruginosa* [28,29,30]. In respiratory epithelia, metformin is known to inhibit *P. aeruginosa* by increasing claudin-1 production and occluding protein abundance. Another study reported that metformin enhanced the innate immunity and resistance to *P. aeruginosa* infection in mice through the activation of the p38 mitogen-activated protein kinase (MAPK) pathway [31]. Although we have only observed this flora change, it has been found that metformin may affect the regulation abundance of *Pseudomonas* via the aforementioned mechanisms.

We also demonstrated the relationships between the alternation of gut microbiota after using metformin and GI symptoms, including diarrhea. Diarrhea has been said to be a more common adverse abdominal symptom caused by metformin compared with constipation [18], but the present study showed that constipation was more severe (5.9 ± 2.2) than diarrhea (3.3 ± 1.7) after four weeks of medication. The reason for this could be the influence of higher constipation scores at the baseline.

Changes in the gut microbiota composition were associated with several abdominal symptoms. Considering the genera that affected changes in these symptoms, we found several relationships in the subgroup analysis. We identified a negative relationship between the genus *Parabacteroides* and symptoms of abdominal pain and regurgitation. Furthermore, the genus *Bifidobacterium* had a negative relationship with regurgitation. *Parabacteroides* is known to be more abundant in patients with Crohn’s disease [32] and functional constipation [33] than in healthy controls. A previous study demonstrated that treatment with orally administered live *Parabacteroides distasonis* dramatically improved the clinical parameters of acute colitis by decreasing the TNF-α production of macrophages [34]. Therefore, abdominal pain and reflux may be exacerbated by a decrease in *Parabacteroides*. *Bifidobacterium* is generally recognized as a traditional probiotic and several studies have shown the benefits of this bacterium [35,36,37,38]. This genus has been associated with modulations in the immune reaction and antagonistic action toward pathogens through short-chain fatty acid (SCFA) production. Furthermore, *Bifidobacterium bifidum* has been shown to attach to stomach cells and promote the production of mucins, which improves the physical gastric barrier [35,36]. Furthermore, in vivo experiments have also shown that *B. bifidum* regulates the NF-κB signaling pathways [36]. The synergistic effects of these mechanisms can relieve abdominal symptoms and improve the symptoms of gastroesophageal reflux disease. Therefore, the frequency of common upper GI symptoms, including regurgitation, is considered to decrease with an increasing abundance of *Bifidobacterium*.

We did not observe a significant change in diarrhea symptoms before and after taking metformin, but we did detect changes in the gut microbiota in those cases. Our study revealed a positive relationship between diarrhea and *Tyzzerella*, which belongs to the *Lachnospiraceae* family within the Firmicutes phylum. *Tyzzerella* has been characterized as a genus that predisposes hosts to diarrhea [37]. Moreover, a higher abundance of *Tyzzerella* is correlated with a higher risk of CVD [38] and has been associated with dietary quality [39]. Therefore, there is a possibility that people with more severe *Tyzzerella*-related diarrhea have an increased risk of developing CVD. Further study is required to clarify the link between diarrhea and the CVD risk. On the other hand, *Blautia* are known to have protective effects on the intestinal epithelium by producing SCFAs [37]; their beneficial effects regarding diarrhea were also displayed in this study. Unfortunately, there have been few reports on the association between diarrhea and *Holdemanella* and *Oscillibacter*.

It is important to mention certain limitations with the current study. First, we did not evaluate each dietary habit or consider the effect of diet on the gut microbiota. Second, we observed gut alternations within the limited duration of the study period of four weeks and the limited number of subjects. Moreover, to confirm the effect of metformin in T2DM patients, it is desirable to observe the change after the cessation of taking metformin or include age-matched healthy control subjects for comparison. Then, the effects metformin has on the gut microbiota and abdominal symptoms will become clearer. Third, because the GSRS is self-administered, the possibility of self-reporting bias was undeniable. Lastly, our study had an open-label and single-armed design.

In summary, the whole-group analysis showed that the composition of gut microbiota in Japanese individuals with T2DM did not change significantly after taking metformin for four weeks. However, in a subgroup analysis, which excluded those using medications that might affect the gut microbiota, the gut microbiota was partially changed and the abdominal symptoms accompanied by metformin usage may be associated with gut microbiota in these individuals. Further research is needed to investigate and reveal the mechanisms underlying the alternation of, and the relationship between, changes in the gut microbiota and abdominal symptoms in Japanese people with T2DM after taking other diabetes medications, as well as to find methods for preventing abdominal symptoms due to metformin usage from the microbiota perspective.

## 4. Materials and Methods

### 4.1. Study Population 

**Figure 7:**
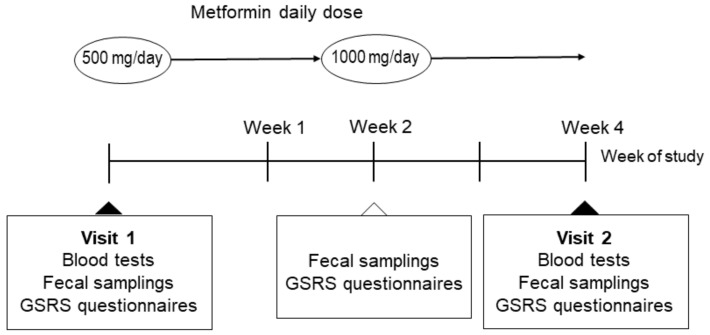
Study design.

This was a two-center, a quasi-experimental study. Between October 2018 and July 2019, we enrolled 20 men and 11 women that were 20−75 years old for HbA_1c_ < 63 mmol/mol (8.0%) trials at Kyoto Prefectural University of Medicine (Kyoto, Japan) and Kajiyama clinic (Kyoto, Japan). Signed informed consent was obtained from all subjects who provided specimens. The study design is shown in Figure 7. The participants took 500 mg of metformin per day for two weeks and then took 1000 mg per day for two weeks, according to a medical package insert. Fecal samples were collected before, after two weeks, and after four weeks of medication. Blood samples were collected before and after four weeks of medication.

We did not include individuals whose estimated glomerular filtration rate (eGFR) was <60 mL/min/1.73 m^2^; those who changed their oral hypoglycemic medications; those who consumed a GLP-1 agonist [3] or antibiotics less than three months before enrollment; nor individuals diagnosed with heart failure, liver failure, or gastrointestinal diseases (Crohn disease, celiac disease, ulcerative colitis, short bowel syndrome, or diverticulosis). During the study period, participants were ordered not to change their lifestyle and/or diet.

### 4.2. Ethical Considerations

This study was approved by the ethics committee of Kyoto Prefectural University of Medicine (approval number ERB-C-1166-2) and undertaken following the Declaration of Helsinki. To protect the confidentiality of participants, personally identifiable data was detached and medical data stored in a database was protected with a password. Medication data were also collected for diabetes, probiotics, proton pump inhibitor (PPI), and H2 blocker medication.

### 4.3. Data Collection and Variables

The BMI, HbA_1c_, systolic and diastolic blood pressure, and GSRS and Bristol Stool Form Scale scores were all examined. HbA_1c_ and creatinine were measured using the subjects’ venous blood samples. The eGFR was calculated using the Japanese Society of Nephrology equation: eGFR = 194 × Cre^−1.094^ × age^−0.287^ (mL/min/1.73 m^2^) (× 0.739 for women) [40]. 

### 4.4. Bacterial DNA Extraction From Feces and DNA Sequence Analysis

The extraction of DNA was performed using a previous method [41]. Twenty milligrams of feces were centrifuged (14,000× *g*) after washing them three times in 1.0 mL of PBS. Three hundred milligrams of glass beads (diameter: 0.1 mm) and 500 µL of buffer-saturated phenol was added to the pellets that were resuspended in 450 µL of extraction buffer (100 mM Tris-HCl, 40 mM EDTA; pH 9.0) and 50 µL of 10% sodium dodecyl sulfate and vortexed vigorously. Then, 400 µL of the supernatant was extracted using phenol-chloroform after centrifugation at 14,000× *g* for 5 min and 250 µL of the supernatant was injected to the isopropanol precipitation. Finally, the DNA was suspended in 1.0 mL of Tris-EDTA buffer. A meta-analysis of the bacterial 16S rDNA sequences in the feces was performed using a previous method [42] with minor modifications. The amplicon of the V3–V4 region of 16S rDNA, which were amplified using a Veriti thermal cycler (Thermo Fisher Scientific, Waltham, MA, USA), was purified using AMPure XP magnetic beads (Beckman Coulter, Brea, CA, USA). Dual eight-base indices (Nextera XT Index kit, Illumina, San Diego, CA, USA) were used for the PCR of the multiplex sequencing. After purification, the purified barcoded library was measured fluorometrically using a QuantiT PicoGreen ds DNA Assay Kit (Invitrogen, Paisley, UK), and the same volume of samples were saved. The library pool (10 pM) was mixed with 40% PhiX control DNA to a final concentration of 10 pM. Sequencing was carried out using a MiSeq platform using a MiSeq Reagent Kit v2 (Illumina). Quality checks confirmed that the purity (Optical Density (OD)260/OD280) of the extracted DNA was greater than 1.8 and the electrophoresis after each PCR confirmed that the bands of interest were clearly visible. Furthermore, the pooled sample library was quantified using Pico green before sequencing with the Miseq platform, and after target DNA-specific quantification with Qubit 4 Fluorometer, Phix control DNA was added and sequenced. Moreover, the percentage of Quality Score 30 was greater than the required 80% and the density of clusters (K/mm^2^) on the flow cell was confirmed to be less than 1000. We also confirmed that the percentage of sequenced reads of the Phix control DNA that spiked in the sample DNA did not deviate significantly from the percentage added to the number of reads obtained.

### 4.5. Microbiota Analysis

The sequence analysis produced 3,692,171 high-quality reads from 93 fecal samples. The selection of reliable sequences, construction of operational taxonomic units (OTUs), and taxonomy assignment was carried out using the Quantitative Insights into Microbial Ecology (QIIME) pipeline (http://qiime.org/) [43]. In brief, for each sample, 50,000 raw reads were randomly gathered from the Miseq raw sequence files, and merging of paired-end reads was carried out using fastq-join with the default setting. As a consequence, reliable sequences were obtained by removing sequence reads with an average quality value of <25 and after checking chimera reads. Moreover, the OTUs were constructed by clustering with a 97% identity threshold after randomly choosing 5000 reliable sequence reads for each sample; then, the taxonomy assignment to the 16S bacterial rRNA database was carried out using UCLUST with a ≥97% identity. Principal coordinates analysis (PCoA) was used to reduce the dimensionality of the resulting distance matrix to compare the differences in the overall bacterial gut microbiota structure.

### 4.6. Statistical Analysis

All statistical analyses were performed using the R statistical software version 3.1.3.25 and JMP 13.2 software (SAS Institute Inc., Cary, NC, USA). Data are shown as means ± standard deviation. Statistical significance was set at *p* < 0.05.

To evaluate the alpha diversity of the microbiota in the samples, the Shannon index, observed OTUs, Chao1, and abundance-based coverage estimator (ACE) were measured using paired *t*-tests. The beta diversity was evaluated by computing the weighted and unweighted UniFrac distances between samples [44]. The beta diversity was analyzed using permutational multivariate analysis of variance (PERMANOVA) for the comparison of gene similarity.

A comparison of each taxon of the gut microbiota was investigated at the phylum and genus levels. The differences in the relative abundances of phyla before and after four weeks of medication, and the ratio of the Firmicutes and Bacteroidetes abundances, which is associated with obesity [45], were evaluated using paired *t*-tests. Furthermore, the relative abundance of the bacterial genera before and after four weeks of medication was also evaluated using paired *t*-tests and the WAD method in R, which can evaluate the genes based on their higher expression, higher weights, or fold change [46]. We chose the top 20 most abundant genera for the graphs. LEfSe analysis was used to detect features that were represented differently between the groups. To find significantly differential taxa, the Kruskal–Wallis sum-rank test was performed, and to evaluate the effect size of these differences, the identified taxa were further subjected to a linear discriminant analysis (LDA). Significant differences were set at *p*-values < 0.05 using the Benjamini–Hochberg procedure [47] and a logarithmic LDA score threshold of 2.0 [48]. 

Subanalyses were also performed. Because some diabetes medications, such as α-glucosidase inhibitors [3], dipeptidyl peptidase IV inhibitors [6,49], PPIs [50], and H2 blockers [51] have been reported to affect the gut microbiota, we also investigated the effect of metformin on the gut microbiota of patients (labeled the subgroup) who did not use these medications.

Lastly, the differences in the blood composition and the GSRS and Bristol Stool Form Scale scores before and after medication were evaluated using paired *t*-tests (for the subgroup). In addition, we investigated the relationship between changes in the GSRS score and the proportion of genera using Spearman’s correlation coefficient.

## Figures and Tables

**Table 1:** Characteristics of study subjects.

Variables	Total	Subgroup
*n*	31	21
Age (years)	63.3 ± 9.5	60.1 ± 9.8
Male	20 (64.5)	15 (71.4)
Body mass index (kg/m^2^)	23.3 ± 3.2	24.1 ± 3.0
SBP (mmHg)	126.2 ± 14.8	127.9 ± 14.6
DBP (mmHg)	71.5 ± 11.7	73.4 ± 10.9
PPG (mmol/L)	7.49 ± 1.63	6.95 ± 1.09
Hemoglobin A_1c_ (mmol/mol)	55.4 ± 7.9	53.7 ± 6.1
Hemoglobin A_1c_ (%)	7.06 ± 0.56	7.06 ± 0.56
eGFR (mL/min/1.73 m^2^)	79.7 ± 13.7	80.1 ± 14.9
Duration of diabetes, (years)	11.5 ± 11.0	8.3 ± 8.3
Diabetic microangiopathy		
Distal symmetric polyneuropathy	2 (6.5)	0 (0)
Retinopathy	3 (9.7)	2 (9.5)
Nephropathy	4 (12.9)	3 (14.3)
H2 blocker	1 (3.2)	-
Proton pump inhibitor	4 (12.9)	-
α-glucosidase inhibitor	1 (3.2)	-
DPP4 inhibitor	9 (29.0)	-

Data are expressed as the number (percentage) and mean ± standard deviation. Subgroup: subjects that did not use an H2 blocker, a proton pump inhibitor, an α-glucosidase inhibitor, or a DPP4 inhibitor; SBP: systolic blood pressure; DBP: diastolic blood pressure; PPG: postprandial plasma glucose; H2 blocker: histamine H2-receptor blocker; DPP4: dipeptidyl peptidase IV.

**Table 2:** Self-reported Gastrointestinal Symptom Rating Scale (GSRS) and Bristol Stool Form Scale scores before and after taking metformin.

Total	Baseline	After 2 Weeks	After 4 Weeks	*p*-Value
Hemoglobin A1c (mmol/mol)	55.4 ± 7.9	-	52.5 ± 6.6	0.0003
Hemoglobin A1c (%)	7.2 ± 0.7	-	7.0 ± 0.6	
Total score	20.3 ± 5.8	21.0 ± 5.4	22.8 ± 7.1	0.04
Subscale				
Abdominal pain	3.6 ± 1.2	3.9 ± 1.7	3.9 ± 1.6	0.24
Regurgitation	2.7 ± 1.6	2.8 ± 1.5	2.8 ± 1.7	0.69
Dyspepsia	6.0 ± 2.1	6.0 ± 1.9	6.8 ± 2.7	0.04
Diarrhea	2.9 ± 1.3	3.2 ± 1.4	3.3 ± 1.7	0.34
Constipation	5.0 ± 2.2	5.5 ± 2.5	5.9 ± 2.2	0.05
Bristol Stool Form Scale	3.7 ± 0.9	3.9 ± 1.0	3.9 ± 1.4	0.44
**Subgroup**	**Baseline**	**After 2 Weeks**	**After 4 Weeks**	***p*-Value**
Hemoglobin A1c (mmol/mol)	53.7 ± 6.1	-	52.3 ± 6.4	0.0005
Hemoglobin A1c (%)	7.1 ± 0.6	-	6.9 ± 0.6	
Total score	20.0 ± 6.0	20.3 ± 4.6	22.6 ± 7.7	0.12
Subscale				
Abdominal pain	3.5 ± 1.1	3.7 ± 1.3	3.8 ± 1.7	0.34
Regurgitation	2.7 ± 1.7	2.8 ± 1.6	2.9 ± 2.0	0.45
Dyspepsia	5.9 ± 2.0	5.9 ± 1.9	6.7 ± 2.9	0.16
Diarrhea	3.1 ± 1.4	3.2 ± 1.4	3.3 ± 1.7	0.83
Constipation	4.7 ± 1.9	5.2 ± 2.1	5.9 ± 2.3	0.03
Bristol Stool Form Scale	3.7 ± 0.9	3.9 ± 1.0	3.9 ± 1.4	0.12

A paired *t*-test between the baseline and after 4 weeks of medication was performed. Subgroup: subjects that were not using an H2 blocker, a proton pump inhibitor, an α-glucosidase inhibitor, and a DPP4 inhibitor; H2 blocker: histamine H2-receptor blocker; DPP4: dipeptidyl peptidase IV.

## Abbreviations

T2DM	Type 2 diabetes mellitus
GSRS	Gastrointestinal Symptom Rating Scale
CVD	Cardiovascular disease
GLP-1	Glucagon-like peptide 1
GI	Gastrointestinal
QOL	Quality of life
BMI	Body mass index
HbA_1c_	Hemoglobin A_1c_
OTU	Operational taxonomic unit
ACE	Abundance-based coverage estimator
PCoA	Principal coordinates analysis
PERMANOVA	Permutational multivariate analysis of variance
WAD	Weighted average distance
LEfSe	Linear discriminant effect size
MAPK	Mitogen-activated protein kinase
SCFAs	Short-chain fatty acids
eGFR	Estimated glomerular filtration rate
PPI	Proton pump inhibitor
OD	Optical Density
QIIME	Quantitative Insights into Microbial Ecology
LDA	Linear discriminant analysis
